# Dentition patterns and molecular diversity of *Mastophorus muris* (Gmelin, 1790) (Nematoda: Spiruroidea) support a host-associated subdivision

**DOI:** 10.1007/s00436-024-08259-1

**Published:** 2024-06-10

**Authors:** Jenny Jost, Jörg Hirzmann, Ľudovít Ďureje, Denny Maaz, Peer Martin, Thomas Stach, Emanuel Heitlinger, Víctor Hugo Jarquín-Díaz

**Affiliations:** 1 Max-Delbrück-Center for Molecular Medicine in the Helmholtz Association (MDC), Robert-Rössle-Str. 10, 13125 Berlin, Germany; 2 Experimental and Clinical Research Center, a cooperation between the Max-Delbrück-Center for Molecular Medicine in the Helmholtz Association and the Charité—Universitätsmedizin Berlin, Berlin, Germany; 3 Charité—Universitätsmedizin Berlin, corporate member of Freie Universität Berlin and Humboldt-Universität Zu Berlin, Experimental and Clinical Research Center, Lindenberger Weg 80, 13125 Berlin, Germany; 4 Department of Molecular Parasitology, Institute for Biology, Humboldt University Berlin (HU), Philippstr. 13, Haus 14, 10115 Berlin, Germany; 5 Research Group Ecology and Evolution of Molecular Parasite-Host Interactions, Leibniz-Institut for Zoo and Wildlife Research (IZW) Im Forschungsverbund Berlin E.V., Alfred-Kowalke-Straße 17, 10315 Berlin, Germany; 6 Institute of Parasitology, Justus-Liebig-University Gießen, Schubertstr. 81, 35392 Gießen, Germany; 7 Research Facility Studenec, Institute of Vertebrate Biology, Czech Academy of Sciences, Květná 8, 603 65 Brno, Czech Republic; 8 Institute for Parasitology and Tropical Veterinary Medicine, Freie University Berlin (FU), Robert-Von-Ostertag-Straße 7, 14163 Berlin, Germany; 9 Comparative Electron Microscopy, Institute for Biology, Humboldt University Berlin (HU), Philippstr. 13, Haus 14, 10115 Berlin, Germany

**Keywords:** Nematoda, *Mastophorus muris*, *Mus musculus*, Scanning electron microscopy, Phylogeny

## Abstract

*Mastophorus muris* (Gmelin, 1790) is a globally distributed parasitic nematode of broad range mammals. The taxonomy within the genus *Mastophorus* and the cryptic diversity among the genus are controversial among taxonomists. This study provides a detailed morphological description of *M. muris* from *Mus musculus* combined with a molecular phylogenetic approach. Moreover, descriptions and molecular data of *M. muris* from non-*Mus* rodents and wildcats complement our findings and together provide new insights into their taxonomy. The analysis of *M. muris* was based on light microscopy and scanning electron microscopy. The morphological description focused on the dentition pattern of the two trilobed pseudolabia. Additionally, we described the position of the vulva, arrangement of caudal pairs of papillae, spicules and measured specimens from both sexes and the eggs. For the molecular phylogenetic approach, we amplified the small subunit ribosomal RNA gene and the internal transcribed spacer, and the cytochrome *c* oxidase subunit 1. *Mastophorus* morphotypes based on dentition patterns and phylogenetic clustering indicate a subdivision of the genus in agreement with their host. We recognize two groups without a change to formal taxonomy: One group including those specimens infecting *Mus musculus*, and the second group including organisms infecting non-*Mus* rodents. Our genetic and morphological data shed light into the cryptic diversity within the genus *Mastopohorus.* We identified two host-associated groups of *M. muris.* The described morphotypes and genotypes of *M. muris* allow a consistent distinction between host-associated parasites.

**Supplementary Information:**

The online version contains supplementary material available at 10.1007/s00436-024-08259-1.

## Introduction

*Mastophorus muris* (Gmelin, 1790) is an euryxenous nematode belonging to the superfamily Spiruroidea (Spirocercidae: Mastophorinae), with a heteroxenous life cycle (Quentin 1970). Intermediate hosts are arthropods of the orders Orthoptera, Dermaptera, Blattodea and Siphonaptera (Grzybek et al. 2015; Quentin 1970). Different species of small rodents (Chitwood 1938; Skrjabin 1961; Rojas and Digiani 2003; Grzybek et al. 2015; Julius et al. 2018; Neupane et al. 2020) including *Mus musculus* (Chitwood 1938; Skrjabin 1961; Kataranovski et al. 2008; Baird et al. 2012) are described as definitive hosts but felines (Skrjabin 1961; Torres et al. 1998), canines (Chitwood 1938; Skrjabin 1961) and even marsupials (Skrjabin 1961; Smales 1995; Smith and Kinsella 2011) have also been reported as hosts.

Within the genus *Mastophorus*, a subdivision into varieties associated with certain hosts has been controversially discussed, and different morphological features have been proposed for this classification (Chitwood 1938; Wertheim 1962; Rojas and Digiani 2003). A major contribution to the classification of *Mastophorus* was provided by Chitwood (1938), who suggested a subdivision of the genus into two varieties based on a single morphological characteristic and host preference: (1) *M. muris* var. *muris* (large denticles, *Rattus norvegicus* and *Felis catus*) wherein *Mus* specimens (intermediate in teeth length) were included for simplicity and (2) *M. muris* var. *ascaroides* (smaller denticles, Geomyoidae, Cricetidae and *Canis latrans*) (Chitwood 1938).

The genus *Mastophorus* has a complex taxonomic relationship with the closely related genus *Protospirur*a (Chitwood 1938; Read and Millemann 1953; Skrjabin 1961; Wertheim 1962; Smales 1995; Rojas and Digiani 2003). The two genera of the superfamily Spiruroidea are morphologically distinguishable based on two traits: the nature of the dentition on the pseudolabia and the proximal position of the vulva with regard to the middle of the body (Chitwood 1938). The shape of the pharynx, egg measurements and spicule characteristics have been suggested as additional differentiation characters (Chitwood 1938; Wertheim 1962) as well as ontogenetic characteristics (Quentin 1970). Nevertheless, despite the discrepancies in the classification of *Mastophorus* and *Protospirura* (Rojas and Digiani 2003; Smales et al. 2009), there is a cryptic morphological diversity within the genus *Mastophorus* that has not been fully explored.

Here, we compare *Mastophorus muris* specimens from different hosts (*Mus musculus*, *Felis silvestris silvestris*, *Myodes glareolus* and *Apodemus flavicollis*) to examine the differentiation of the genus in varieties according to their host range. We provide general morphological descriptions and molecular data of specimens from different hosts. To differentiate the specimen from our study, we focused on the detailed description of the dentition pattern besides phylogenetic analyses.

## Material and methods

### Sample collection

A total of 567 house mice (*Mus musculus*) collected in Brandenburg (Germany), during annual field trips in autumn 2016 to 2018, were used for the present study (capture permit No. 2347/35/2014). Mice were dissected for inspection of helminths in the body cavity and gastrointestinal tract. Specimens from each host were individually collected and preserved in 10% Neutral-Buffered Formalin solution for detailed morphological description and in 70% (v/v) ethanol for DNA extraction and stored at room temperature until further analysis. Feces were collected and stored in a 2.5% (w/v) solution of potassium dichromate (K_2_Cr_2_O_7_) at 4 °C until further microscopic observations of parasite eggs.

Four *M. muris* specimens from non-*Mus* rodents were collected in Berlin in 2010, two from the bank vole (*Myodes glareolus*) and two from the yellow-necked mouse (*Apodemus flavicollis*) (one female and one male from each host) (Maaz et al. 2018), stored in 70% (v/v) ethanol and used for morphological description and DNA extraction. Morphological characteristics and extracted DNA from *M*. *muris* specimens collected from the wildcat (*Felis silvestris silvestris*, *N* = 2) were integrated in further analyses.

Voucher specimens for *M. muris* from *Mus* were deposited in the Natural History Museum in Berlin, Germany in the department “Vermes” under specimens numbers E.7635 – E.7639.

### Morphological analysis

Morphological description of specimens (*N* = 125, 78 females and 47 males) was performed following taxonomic keys of parasitic nematodes (Hall 1916; Skrjabin 1961; Chabaud 1975; Sutton 1989). Morphometric data of the length, width and vulva position were recorded with an Olympus SZ61 stereo microscope. To visualize the structure of the spicules, male specimens fixed in ethanol were treated with a potassium hydroxide solution 10% (w/v) for 3 days at room temperature. Eggs were collected in a flotation of feces with a saturated salt solution (Jarquín-Díaz et al. 2019). Micrographs of eggs (*N* = 30, 400 × magnification) and spicules (*N* = 6, 100 × magnification) were taken with an Axioplan Carl-Zeiss light microscope and measured using Adobe Photoshop CC v 14.2.1.

Specimens (*N* = 16, eight females and four males from *Mus*, two females and two males from non-*Mus*) for scanning electron microscopy (SEM) were first fixed in 2% (v/v) paraformaldehyde and 2.5% (v/v) glutaraldehyde in phosphate buffer (pH = 7.4) and then treated with 2% (v/v) osmium tetroxide. They were rinsed in distilled water, dehydrated in an alcohol series, critical point dried in carbon dioxide (BAL-TEC CPD030 Critical Point Dryer) and finally gold-coated (BAL-TEC SCD005 Sputter Coater). The samples were examined using an SEM LEO 1430 (Zeiss) and the associated SmartSEM V06.00 operating software, and the resulting scanning electron micrographs were post-processed using Adobe Photoshop CC v 14.2.1.

### Data analysis

Parasite prevalence, abundance and intensity as defined by Lafferty et al. (1997) were determined. All calculations were performed in R (R Core Team 2008). For prevalence, 95% confidence intervals were calculated using Sterne’s exact method (Rózsa et al. 2000), using the package “epiR” (Stevenson et al. 2018).

### DNA extraction

The morphologically less informative part of a single worm specimen was mechanically disrupted with a micro pestle in 20 µL of nuclease-free water (the other part was saved for morphological assessment). Genomic DNA was extracted using the innuPREP DNA Mini Kit (Analytik Jena AG, Jena, Germany), following the protocol of the manufacturer for tissue samples and rodent tails. Adjustments were made within the lysis step (30 µL proteinase K and 1-h incubation time) and the elution step (adding 50 µL elution buffer with one repetition). Modifications to lysis conditions were applied (30 µL of protein kinase and 1 h at 50 °C incubation). The DNA was eluted twice in a final volume of 50 µL.

### PCR amplification

Previously published primer pairs specific for nematodes were used, which target partial sequences of the nuclear genome: 18S rDNA (18S) (Floyd et al. 2005), the internal transcribed spacer (ITS) region (including ITS1, 5.8S and ITS2) (Gasser and Hoste 1995) and partial sequence of the mitochondrial cytochrome *c* oxidase subunit 1 (CO1) gene (Bowles et al. 1993; Casiraghi et al. 2001; Blaxter 2004). In addition, a primer pair aiming to complete the 18S region was designed, based on the sequence of *M. muris* from wildcat (MG818763) and the already amplified regions using Geneious R6 v. 6.1.8 (https://www.geneious.com) (Kearse et al. 2012) (Supplementary Table 1). PCR was performed using ThermoScientific DreamTaq DNA Polymerase (Thermo Fisher Scientific Inc.) as detailed in Supplementary Table 2. Amplified PCR products with the expected size were purified with the SAP-Exo Kit (Jena Bioscience GmbH, Jena, Germany), following the specifications of the manufacturer and sequenced in both directions by LGC (LGC Genomics GmbH, Berlin, Germany).

Consensus sequences for each gene and specimen were generated by assembly of forward and reverse sequencing reads, and further alignment of overlapping regions between amplicons was generated with different primer pairs by assembling reads with reference sequences (18S MG818763 and CO1 AJ537512) in Geneious. The nearly complete 18S region (~ 1670 bp), sequences with ~ 850 bp for the CO1 and ~ 1000 bp for ITS for *Mastophorus* specimens from *Mus* and *Apodemus* (none for ITS region) were submitted to NCBI GenBank with the accession numbers: 18S [MN086286–MN086291], CO1 [MK867474–MK867480] and ITS [MK829001–MK829007]. Genetic data of *M. muris* from *Myodes* could not be obtained.

### Phylogenetic analyses

Datasets for each gene were generated individually, including all available sequences in the GenBank from *Mastophorus* and closely related organisms only from the superfamily Spiruroidea. Close related sequences MT512662 (*Protospirura* sp.—CO1), KT894811, KT894812 (*Protospirura numidica*—18S), JQ771745 and JQ771746 (Spiruridae sp.—18S) were excluded from any phylogenetic analysis due to lack or questionable morphological identification and taxonomic assignment. Relevant available information from those sequences regarding host and geographical origin, length and authors are listed in Supplementary Table 3*.* The individual sequence datasets for 18S, CO1 and ITS were aligned using the profile-to-profile method implemented within the R package DECIPHER v.2.22 (Wright 2015, 2020). The CO1 dataset was aligned using the codon-based algorithm. For all datasets, missing data was indicated in the sequences as “?”. The R package Phangorn v. 2.11.1 (Schliep 2011; Schliep et al. 2016) was used to determine the substitution model with the best fit for each alignment (appropriated sequence evolution models for each dataset—18S: TPM2 + I, ITS: HKY + G, CO1: TIM3 + G). Phylogenetic estimation was done by maximum likelihood (ML) implemented in Phangorn v. 2.11.1 (Schliep 2011; Schliep et al. 2017) with 1000 bootstrap replicates and Bayesian inference (BI) implemented in MrBayes v. 3.2.7 (Huelsenbeck and Ronquist 2001; Ronquist and Huelsenbeck 2003) using two heated and two cold chains sampled every 100 generations for a total of 1,000,000 generations with an average standard deviation of split frequencies below 0.01 and using a relative burn in of 25% for diagnostic. Phylogenetic inference accounted for missing data.

For all genetic analyses, sequences from *Dirofilaria* were used as an outgroup for rooting (Supplementary Table 3). Visualization and editing of the phylogenetic trees were conducted in Figtree v.1.4.4 (http://tree.bio.ed.ac.uk/software/figtree/) and Inkscape v. 0.92 (https://inkscape.org).

## Results

### Occurrence of *Mastophorus muris* in house mice

A total of 207 M*. muris* were collected from 21 of 567 investigated mice, corresponding to a prevalence of 3.7% (95% confidence interval (CI): 2.35–5.62). Infected mice were from 14 different localities in North-Eastern Germany. In all cases, worms were located in the stomach of the host. The maximum intensity was 46 M*. muris* in one host, mean intensity was 9.86 (95% CI: 6.14–16.27) and the abundance was 0.37 (95% CI: 0.19–0.75).

### Morphological descriptions of *M. muris* from *M. musculus*

**Fig. 1:**
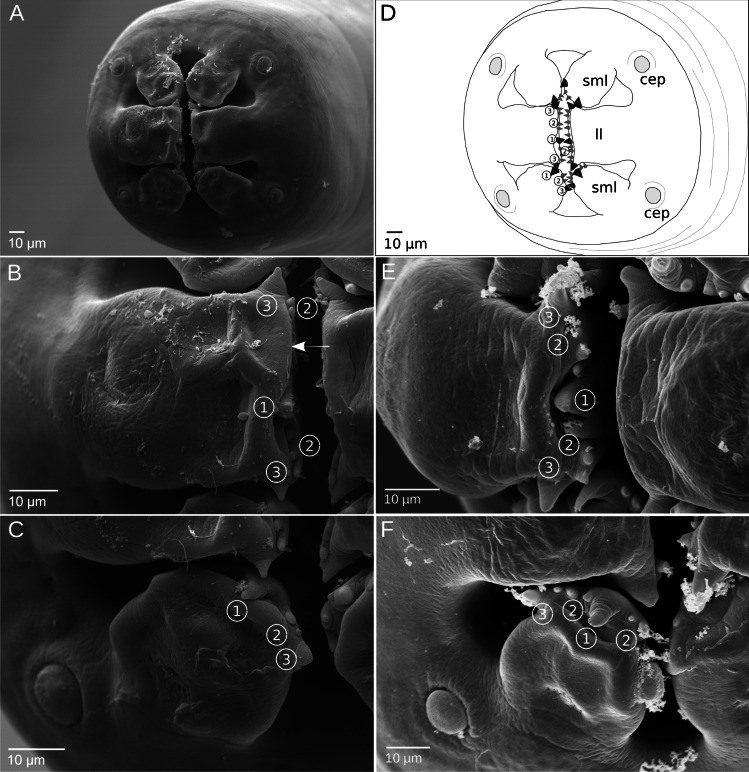
Scanning electron micrographs of *Mastophorus muris* specimens from *Mus musculus*. **A** En face view of the mouth opening, schematized in (**D**) showing two trilobed pseudolabia each consisting of one lateral (ll) and two submedian (sml) lobes, with two cephalic papillae (cep) located at its base. Note different shapes of lobes: lateral lobes are square-shaped (**B, E**), framed by two smaller, slender submedian lobes (**C, F**). Scheme (**D**) illustrates the visible dentition (marked in **B**, **C**, **E** and **F**) at the distal margin of each lobe. Dentition consists of a large central tooth (1), smaller median denticles (2) and a large tooth on each edge (3). Variations of the dentition of the pseudolabia are shown from two specimens (specimen AA0256 in B and C, AA0351 in E and F; see Supplementary Table 4 for list of samples and further details). A separation in two membranes is visible at the toothed distal margin of the lateral lobe (**B**). cep—cephalic papilla, ll—lateral lobe, sml—submedian lobe

Morphological observations and measurements for our *Mastophorus* specimens from SEM and light microscopy are summarized in Table 1. The body surface of all specimens shows a transversal striation, which is attenuated towards the anterior end, showing a circular mouth opening surrounded by two trilobed pseudolabia (Fig. 1A, D). Each pseudolabium is composed of one lateral and two submedian lobes. The lateral lobe is large, square-shaped (Fig. 1B, E) and framed by two smaller, slender and more tapered submedian lobes (Fig. 1C, F). Four cephalic papillae are located at the base of the pseudolabia, one pair per labium (Fig. 1A, D). At the distal margin of each lobe, “denticle-like” structures of variable size and irregular shape are visible (Fig. 1B–F). Larger denticles protrude at both edges and in the middle of each lobe (Fig. 1B–F). The denticles are located with different membranes.

**Table 1:** Morphometric and morphological characteristics from newly generated and previously reported *M. muris* specimens and *Protospirura* spp

	*M. muris*	*M. muris*	*M. muris*	*M. muris*	*M. muris*	*M. muris*	*M. muris*	*M. muris*	*P. numidica criceticola*	*P. muricola*
	Present study	Present study	Present study	Present study	Wertheim 1962	Rojas and Digiani 2003	Chitwood 1938	Chitwood 1938	Quentin et al. 1968	Smales et al. 2009
Host	*Mus musculus*	*Myodes glareolus*	*Apodemus flavicollis*	*Felis silvestris silvestris*	*Rattus spp.*	*Graomys griseoflavus*	*Mus musculus*	*Rattus norvegicus*	*Zygodontomys lasiurus*	*Acomys dimidiatus*
Locality	Germany	Germany	Germany	Germany	Israel	Argentina	USA	USA	Brazil	Egypt
Number of individuals*	F = 78, M = 47	F = 1, M = 1	F = 1, M = 1	F = 2	F = 37, M = 25	F = 12, M = 12	-	-	-	F = 17, M = 15
Dentition pattern**	[1–(2 + *n*) –1– (2 + *n*)–1]	[(3–1–3)] [(7 up to 9)] [(3–1–3)]		[(3 or 7)–1– 3 or 7)]	[v.n.–1–v.n.]	[1–3–1–3–1]	(3/5/7 or 9)	(3/5/7 or 9)	[2][4][2]	[2][2, plate-like][2]
Female										
Length	9.53–39.16 (26.43)	27.04	23.63	23.8	23.0–87	15.7–24.3 (19.8)	38	20	50	35–45 (42)
Width	0.30–1.78 (1.13)	0.9	1.13	-	0.7–2.6	-	0.88	0.8	0.760	0.612–1.29 (1.19)
Vulva position***	34.10—42.38%	39.28%	35.38%	31.93%	30–56%	31–44%	37%	-	58%	~ 57%
Eggs										
Length	0.054–0.064 (0.058)	-	-	0.047–0.056	0.051–0.055	0.046–0.076	-	-	0.045	0.049–0.059 (0.056)
Width	0.033–0.036 (0.034)	-	-	0.029–0.030	0.031–0.032	0.019–0.046	-	-	0.033	0.040—0.046 (0.042)
Male										
Length	10.10–27.96 (18.61)	18.99	19.47	-	17.0–56.0	9.0–16.0 (12.6)	16	14	20.0—24.5	19–26 (22.2)
Width	0.38–0.94 (0.69)	0.61	0.78	-	0.4–1.3	-	0.48	0.544	0.600	0.46–0.80 (0.633)
Spicule right										
Length	0.910–1.519 (1.217)	-	-	-	1.15–1.52	0.79–1.4 (0.99)	1.0	1.016	1.000—1.420	0.268–0.43 (0.352)
Width	0.016–0.030 (0.024)	-	-	-	0.034–0.040	0.027–0.05 (0.032)	-	-	0.030	-
Spicule left										
Length	0.639–1.161 (0.974)	-	-	-	0.95–1.30	0.72–1.08 (0.96)	0.88	0.816	0.470	0.29–0.501 (0.411)
Width	0.012–0.026 (0.020)	-	-	-	0.025–0.027	0.013–0.05 (0.021)	-	-	0.075	-
Genital papillae	Four pairs precloacal pedunculate	Four pairs precloacal pedunculate	Four pairs precloacal pedunculate	-	Four pairs precloacal	Four pairs precloacal	-	-	Four pairs precloacal	Four pairs precloacal,
	Two pairs postcloacal pedunculate	Two pairs postcloacal pedunculate	Two pairs postcloacal pedunculate	-	Two pairs postcloacal	Two pairs postcloacal	-	-	Six pairs postclocal (two large, four small)	Six pairs postcloacal (two large, four small)
						One unpaired papilla ant. of the cloaca			One unpaired papilla ant. of the cloaca	One unpaired papilla ant. of the cloaca
	v.n. of distal sessile caudal papillae	v.n. of distal sessile caudal papillae	v.n. of distal sessile caudal papillae		v.n. of small papillae at the tail	v.n. of small papillae at the tail				

All measurements provided as mm range and (mean)

^*^F—female, M—male, number of investigated specimens

^**^Dentition pattern: “[]” corresponds to the number of denticles for one lobe. The trilobed pseudolabia consist of three lobes given as [] [] []; if only one [] is displayed, the same pattern occurs on each lobe. (v.n.—various number)

^***^% of body length from anterior end. The source of the previous morphological data is reported at the top of the columns

At the distal margin of the lateral lobe, the separation into an outer membrane (Fig. 1B) that bears the large denticles at the edges (3) and an inner membrane with the large central tooth (1) and a variable number of smaller denticles (2) is visible (Fig. 1B). The number of smaller denticles varies between lobes and from specimen to specimen (Fig. 1B, C, E, F, Supplementary Table 4). For the *Mastophorus* specimens from *Mus*, a general dentition pattern can be specified with: 1–(2 + *n*)–1–(2 + *n*)–1.

**Fig. 2:**
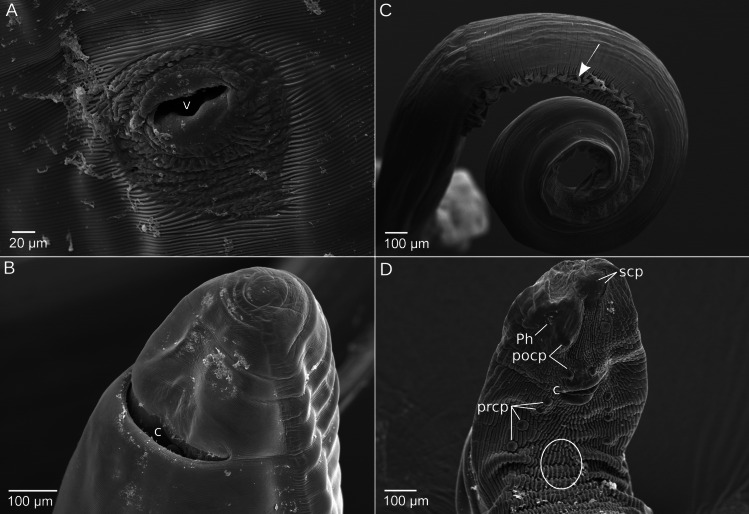
Scanning electron micrographs of female (**A–B**) and male (**C–D**) *Mastophorus muris* specimens from *Mus musculus*. Ventral view shows the vulva located anterior to the middle of the body (**A**). Ventro-lateral view of the female tail showing cloaca and a rounded tip (**B**). Lateral view of the coiled male tail showing a wide caudal ala (indicated with an arrow) (**C**). At the ventral view of the tail longitudinal striations, cuticular modifications (marked with a circle), the cloaca, the phasmidial orifices, distal sessile caudal papillae and six pairs of pre-/postcloacal pedunculate papillae are present (**D**). v—vulva, c—cloaca, prcp—precloacal papillae, pocp—postcloacal papillae, Ph—phasmidial orifices, sessile caudal papillae—scp

**Fig. 3:**
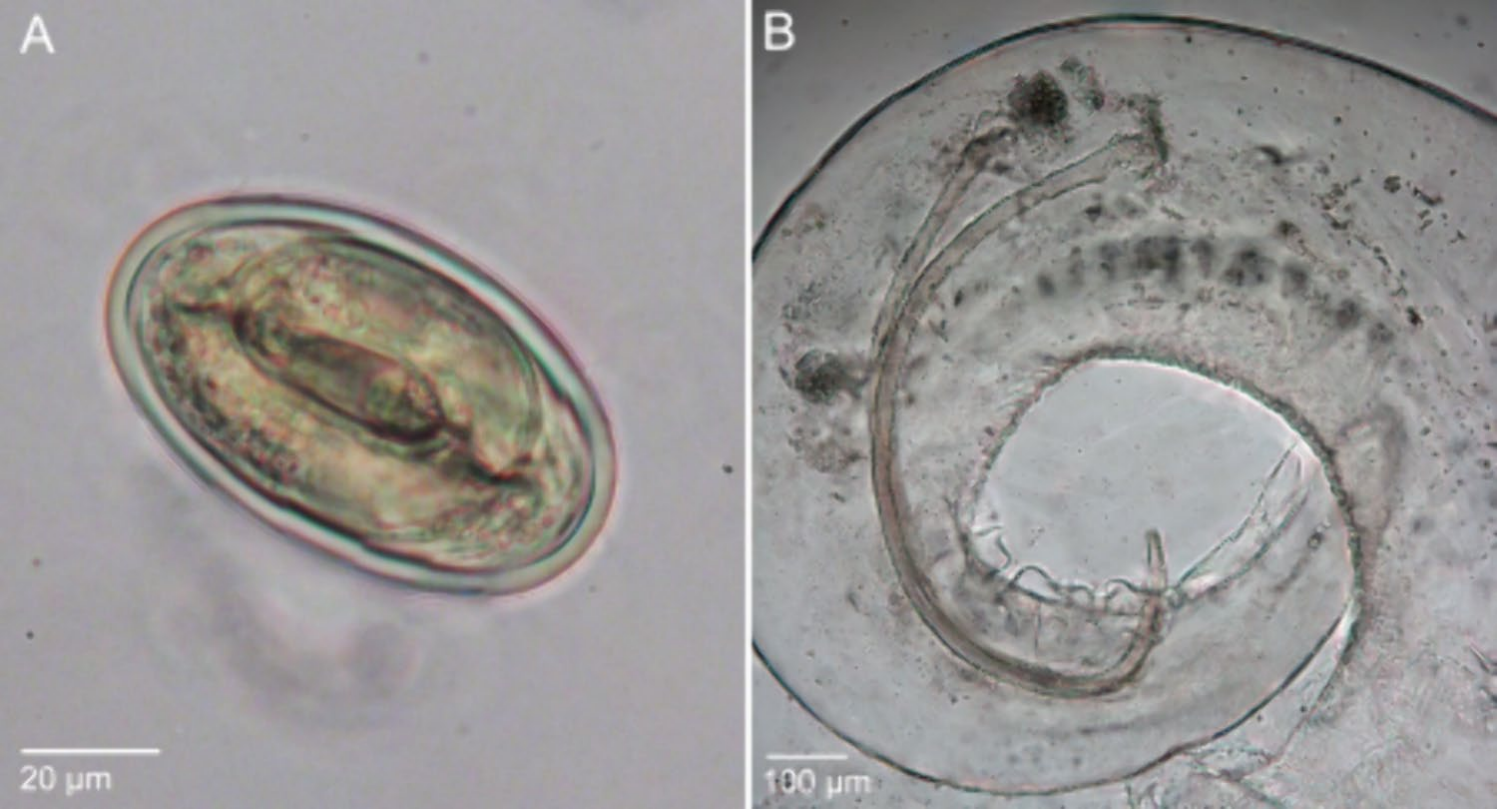
Mature egg (**A**) and the posterior end of a male specimen (**B**) of *Mastophorus muris* from *Mus musculus*, light microscopy. **A** Thick-shelled *Mastophorus muris* egg shows a first-stage larva detected in the floated feces. **B** Lateral view of the coiled tail showing spicules of different length and width (alkaline treatment)

Females (*N* = 78) are 9.53–39.16 (26.43 ± 7.01) mm long and 0.30–1.78 (1.13 ± 0.34) mm wide. The vulva (Fig. 2A) is a transverse fissure located anterior to the middle of the body, in a position around 34.10–42.38% (*N* = 10) of the total body length. The posterior end of female specimens is rounded, and the cloaca can be observed (Fig. 2B). *Mastophorus muris* eggs (*N* = 30, fecal flotations from different hosts) are oval and thick-shelled (Fig. 3A), with a length of 0.054–0.064 (0.058 ± 0.002) mm and a width of 0.033–0.036 (0.034 ± 0.001) mm and contain a first-stage larva.

Males (*N* = 47) are 10.10–27.96 (18.61 ± 3.24) mm long and 0.38–0.94 (0.69 ± 0.13) mm wide. Their posterior end is longer and more coiled compared to female specimens, and has a wide caudal ala (Fig. 2C, indicated with an arrow). The posterior surface is heavily ornamented ventrally with longitudinal striations and cuticular modifications (Fig. 2D). At the posterior end, in total six pairs of pedunculate caudal papillae are present: four pre-cloacal pairs and two post-cloacal pairs (Fig. 2D). At the anterior lip of the cloacal aperture, an unpaired median papilla was observed (Fig. 2D). Additionally, a sessile papillae and phasmidial orifices are observed (Fig. 2D). We observed two unequal spicules become visible (Fig. 3B), the larger right spicule is 0.910–1.519 (1.217 ± 0.151) mm long and 0.016–0.030 (0.024 ± 0.005) mm wide, and the smaller left spicule is 0.639–1.161 (0.974 ± 0.144) mm long and 0.012–0.026 (0.020 ± 0.004) mm wide.

### Morphological description of *M. muris* from non-*Mus* rodents

**Fig. 4:**
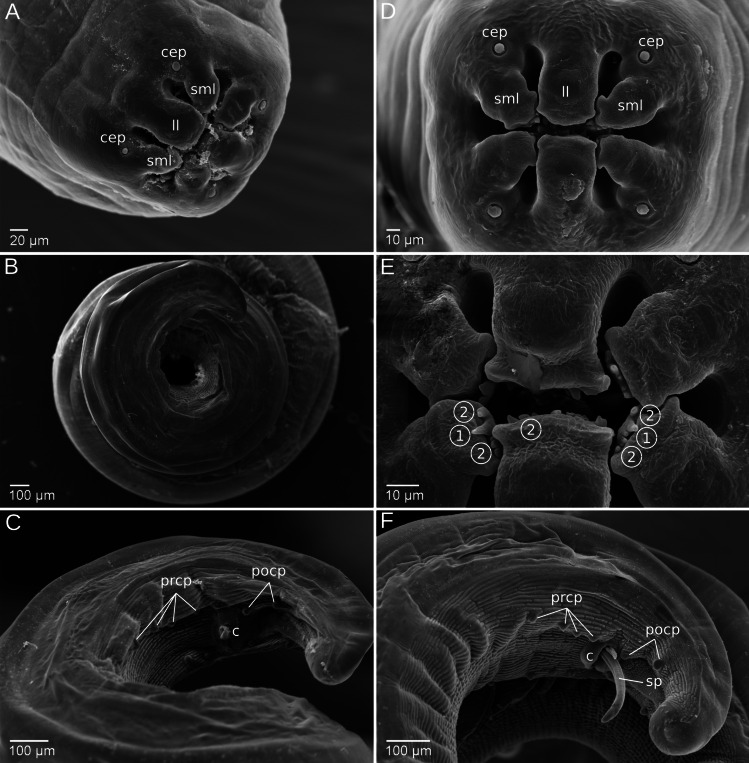
Scanning electron micrographs of *Mastophorus muris* specimens from *Apodemus flavicollis* (**A–C**) and from *Myodes glareolus* (**D–F**). Face view of the mouth opening, which is surrounded by two trilobed pseudolabia each with one lateral (ll) and two submedian (sml) lobes and with four cephalic papillae (cep) located on their base (**A** and **D**). Visible dentition is marked with (1) for a large median tooth and with (2) for smaller denticles, variable in numbers (**E**). A lateral view of the coiled posterior end of male specimens shows the cloaca (c) and six pairs of pre-/postcloacal papillae (prcp/pocp) (**C** and **F**). The extended spicules (sp) are visible in the lateral-ventral view of the tail of one *Myodes* specimen (**F**). c—cloaca, prcp—precloacal papillae, pocp—postcloacal papillae, sp—spicule

One female and one male specimen from *A. flavicollis* and *M. glareolus* were analyzed, using light microscopy and SEM. General structures of the body surface, the apical and posterior region were found to be indistinguishable between specimens from different rodent hosts (Fig. 4). Measurements for body size and vulva position are consistent with the previous descriptions of *M. muris* (Table 1), including our own description of *M. muris* from *M. musculus*.

Dentition pattern could be observed in specimens from *Apodemus* and *Myodes* hosts (Fig. 4A–D). No large denticles were detected at the edge of the lobes (Fig. 4E), and only the smaller submedian lobes show a large central tooth (1) framed by two or three smaller denticles (2) on each side (dentition pattern 3–1–3). The central lobe of the specimen from *Myodes* shows a dentition with seven to nine denticles of unequal medium size (Fig. 4E).

### Morphological description of *M. muris* from *F. silvestris*

From wildcat carcasses, two female *M. muris* were isolated, one from the intestine and one from the pulmonary vessel of the lung. The *Mastophorus* from wildcats had a circular mouth opening and non-compressed pharynx (not shown) and presented the following traits under a light microscope (Supplementary Fig. S1): (1) vulva position at the anterior part of the body (31.93%); (2) dentition with a central big tooth and a variable uneven number of smaller denticles on the trilobed pseudolabia; (3) narrow oval eggs (*N* = 6, 0.053 × 0.030 mm). Morphological traits and measurements were in agreement with previously published data (Table 1).

### Dentition of *M. muris* in comparison to previous reports

**Fig. 5:**
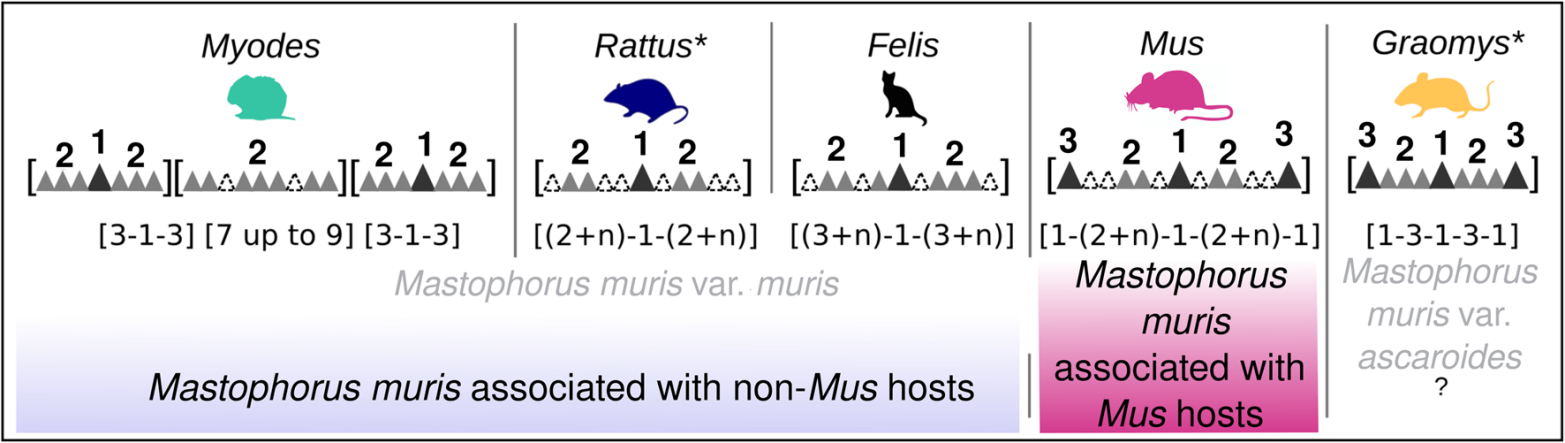
Illustration of the variants of dentition patterns of *Mastophorus muris* obtained from different hosts. The schema illustrates the dentition consisting of a large central tooth (1), various numbers of smaller median denticles (2) and a large denticles on each edge (3) if applicable (dashed lines represent variability). In the line below, the dentition formula for each host is shown, either for all three lobes per pseudolabia [][][] or one representative lobe []. The subdivision of the genus *Mastophorus* by Chitwood is shown in gray and associated with our assignment in *Mastophorus muris* associated with *Mus* (pink) and *Mastophorus muris* associated with non-*Mus* hosts (purple) for specimens from *Felis*, *Myodes* and *Rattus*. * Previous published data for *Rattus* and *Graomys*

Dentition patterns are variable between specimens (Supplementary Table 4). Nevertheless, we were able to generalize the following dentition pattern for specimens from *Mus* hosts per lobe (Fig. 5): a large central tooth (1), large denticles on the edges of each lobe (3) and a variable number of smaller denticles in between (2). This can be expressed as the formula 1–(2 + *n*)–1–(2 + *n*)–1 (the dash separating large denticles and smaller denticles in brackets). In contrast, non-*Mus* specimens (from *Myodes*, *Rattus* and *Felis*) (Wertheim 1962) exhibit a large central tooth (1), a variable number of smaller denticles in between (2) and no large denticles at the edges of each lobe. For *M. muris* from *Graomys* (Fig. 5, Rojas and Digiani 2003) a fixed pattern was reported, always having three smaller denticles between large single denticles on one lobe (1–3–1–3–1).

### Genetic differences between *M. muris* from different hosts

To assess the phylogenetic pattern of *M. muris* from house mice to previously investigated specimens, we inferred phylogenetic trees, using the most commonly reported genetic markers for these nematodes.

**Fig. 6:**
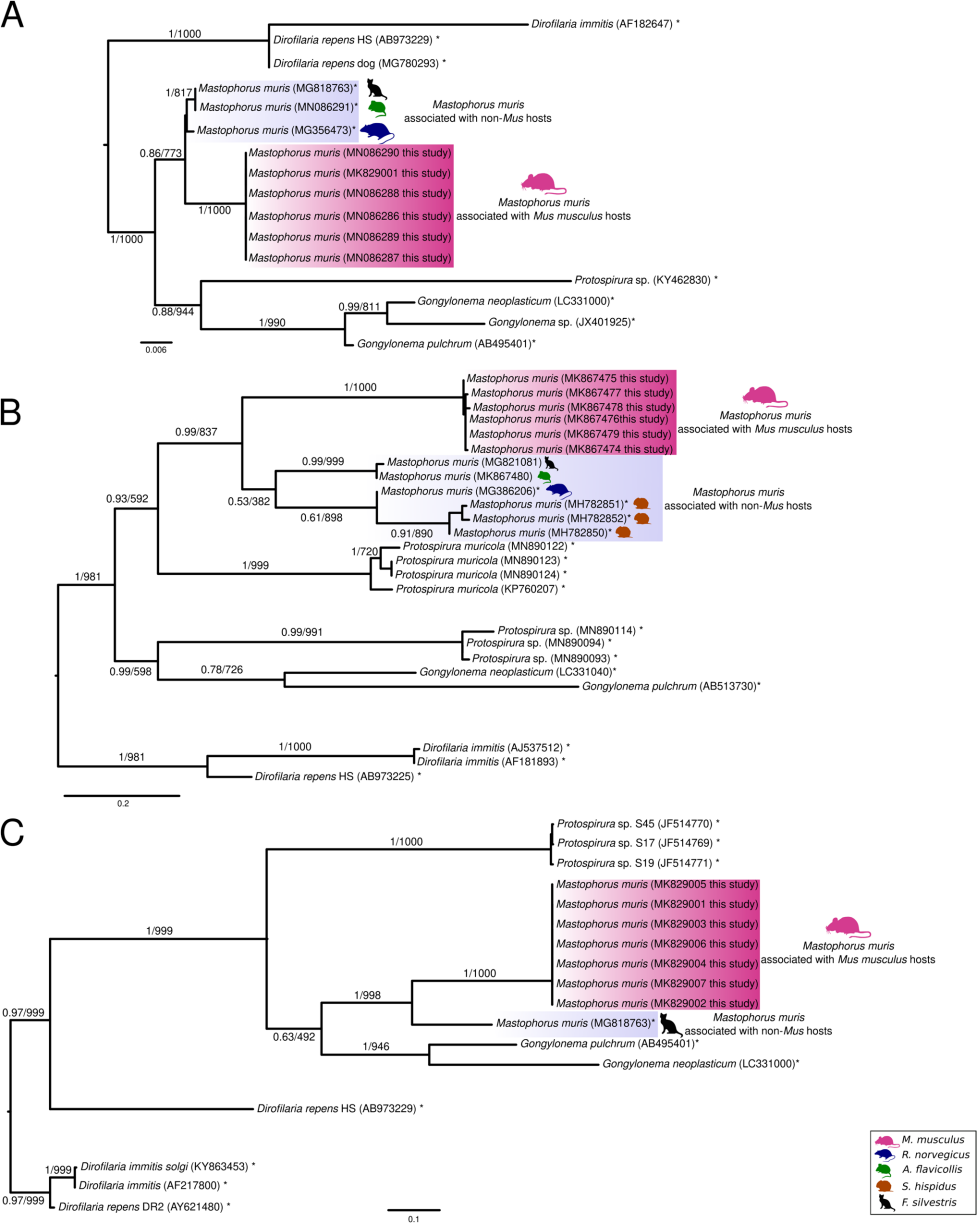
Phylogenetic tree based on 18S (**A**), CO1 (**B**) and ITS (**C**) sequences. Analyses include reference sequences (marked with an (*) asterisk) from Spiruroidea and as outgroup *Dirofilaria* spp. isolates. *Mastophorus* sequences are found in two distinct clades, one with isolates from *Mus* (pink) and a second clade with isolates from wildcat (black), rat (blue), *Apodemus* (green) and *Sigmodon* (brown) colored in purple. Bayesian posterior probabilities followed by bootstrap values are displayed on the branches and the substitution rate per site in the bottom scale bar

A phylogenetic tree for 18S sequences was based on 16 sequences (1692–1748 bp), including *M. muris* and other nematodes (Spirurina) (Fig. 6A). Sequences from specimens from *Mus* (*N* = 6) formed a well-supported clade, separating them from *M. muris* specimens, isolated from *A. flavicollis* (*N* = 1), *F. silvestris silvestris* (*N* = 1) and *Rattus norvegicus* (*N* = 1). *Gongylonem*a sequences were recovered in a clade with the sister group being a sequence deposited as *Protospirura* sp. to GenBank (accession number: KY462830.1). The latter sequence obtained from a specimen isolated from *Mastomys coucha* in South Africa is nearly identical (99%) to a partial (631 bp) 18S sequence of *P. muricola* (KP760162) from a gorilla of Central African Republic, but has a sequence identity of only 95% to *P. numidica* (KT894812, KT894811) not included for our analysis due to its controversial morphological assignment (Costa et al. 2018).

A phylogenetic tree based on CO1 sequences (Fig. 6B) was inferred from 24 sequences (369–858 bp), 10 *Mastophorus* sequences from different hosts (six *Mus*, one *Apodemus*, three *Sigmodon*, one *Felis* and one from a wild rat), 11 further sequences of representatives of the superfamily Spiruroidea and 3 *Dirofilaria* sp. sequences. In agreement with the 18S phylogenetic tree, *M. muris* from house mice formed a distinct clade with high support and sequence identities of 99.6–100% (795 bp). Partial CO1 sequences of *M. muris* isolates from *Apodemus* and *Felis* are 99.6% (801 bp) identical, thus the same *Mastophorus* species. We observed sequence identities of only around 87% (795 bp) between specimens from *Mus* and non-*Mus* hosts (*Felis*, *Apodemus*, *Rattus* and *Sigmodon*).

The phylogenetic tree for ITS sequences (Fig. 6C) was based on 17 sequences (490–1351 bp): five sequences of the superfamily Spiruroidea, one sequence from *M. muris* from wildcat and our six sequences from *Mus* were included.

All markers support a close relationship of *Mastophorus* sequences from *Mus musculus* in one clade, contrasted with a second clade consisting of all other available *Mastophorus* sequences from other hosts. A genetic distinction between two *Mastophorus* groups according to the hosts they were isolated from is overall moderately to well supported.

## Discussion

We provide a detailed morphological description of *M. muris* focused on the dentition pattern of the pseudolabia as a character allowing the distinction of host-associated varieties. We compare this novel morphological distinction with published descriptions and link it with genetic data following the principles of integrative taxonomy (Blaxter 2004; Dayrat 2005; De Queiroz 2007). Genetic data in the present study confirm the phylogenetic placement of *M. muris* varieties as sister group to *Protospirura* sequences available in databases.

A distinction based on morphology between *M. muris* specimens from different hosts was only apparent in the dentition pattern on the lobes of the pseudolabia. We had additionally evaluated the size of the worms, the size of spicules, the lobe substructures of the pseudolabiae, the position of the vulva in females and the arrangement of papillae on the tail of males. The suitability of the dentition pattern as a distinguishing morphological character had been proposed in previous reports (Chitwood 1938; Wertheim 1962; Rojas and Digiani 2003); as a consequence, other morphological characters also suggested in nematodes systematics such as distances to nerve ring, excretory pore and deirids from the cephalic end, the lengths of the pharynx, muscular and glandular esophagus, and tail length etc. were not considered in the present study, which might represent a limitation in our work. However, in contrast to Chitwood (1938) who based subdivision of the genus on the size of denticles, we propose the composition of dentition (pattern of large and small denticles per lobe) as a distinguishing trait.

According to the classification by Chitwood (1938), only one morphological character (size of denticles) allows a subdivision in variants of the genus *Mastophorus.* Within this classification, specimens from *Mus* (with denticles of intermediate size) are subsumed with those described from *Myodes*, *Rattus* and *Felis* into the group *M. muris* var. *muris* (with large denticles) (Chitwood 1938). Specimens from *Graomys* (with smaller denticles) are categorized to *M. muris* var. *ascaroides* (Chitwood 1938). Here, we suggest a subdivision based rather on the composition of the dentition (i.e. based on the presence/absence of the central large or large denticles on the edge of lobes). These characters allow a consistent distinction of two variants: one occurring in *Mus* hosts and the second in non-*Mus* (*Felis*, *Myodes* and *Rattus*) hosts. The classification solely based on the dentition pattern we propose here would render specimen from *Graomys* hosts*,* classified as *M. muris* var. *ascaroides* (Chitwood 1938; Rojas and Digiani 2003), indistinguishable from our *M. muris* from *Mus* hosts. However, the presence of additional unpaired papilla on the posterior end of male specimens from *Graomys* distinguishes these from specimens from *Mus*.

We generalized the dentition for *M. muris* from *Mus musculus* to 1–(2 + *n*)–1–(2 + *n*)–1 per lobe of the pseudolabia and grouped these specimens in *M. muris* associated with *Mus*. All *Mastophorus* specimens from non-*Mus* hosts do not possess large denticles at the edges of pseudolabial lobes. Therefore, we assigned these specimens to *M. muris* associated with non-*Mus* hosts. Specimens of *Mastophorus* from *Graomys* (Rojas and Digiani 2003) represent an exception, because dentition assigns them to *M. muris* associated with *Mus*, but the number of caudal papillae allows a differentiation in this case. We suggest additional research to clarify whether specimens from *Graomys* justify instituting a new *Mastophorus* variant for them.

In conclusion, we found that the dentition pattern is the most reliable morphological character to distinguish host-associated variants of *M. muris*. We support a subdivision of *Mastophorus* into two variants: *M. muris* associated with *Mus* and *M. muris associated* with non*-Mus* hosts (hosts: *Apodemus*, *Felis* and *Rattus*). Previous descriptions of *M. muris* contain numerous traits with high morphological and morphometric variability (Chitwood 1938; Wertheim 1962; Rojas and Digiani 2003). This is a challenge for the potential subdivision of the genus as it is possible that we overlooked variability in the dentition pattern for specimens from non-*Mus* hosts due to our shallow sampling of few specimens. We argue, however, that the variants observed for worms from *Myodes* and *Rattus* hosts fall clearly outside of the variability observed in specimens from *Mus* hosts. The latter were sampled densely in an area overlapping the sampling for the other rodents. *Mastophorus* from the same host but different geographical regions showed low variability based on their partial CO1-sequences which confirmed host association. For example, the *Mastophorus* from *Apodemus* and *Felis* (*Mastophorus* probably from a preyed *Apodemus*) which were collected from geographical regions approx. 600 km apart (Tegel, Berlin and Usingen, Hesse in Germany) showed high identity of their partial CO1 sequences. The same applies to the CO1 sequences of *Mastophorus* isolates from *Sigmodon* which were collected approx. 400 km apart (Piedmont region of Georgia and Costal Plains region of Georgia; Thompson et al. 2019) and the isolates from *Oxymycterus* which were collected approx. 1000 km apart (Ilha Grande, Rio Janeiro and Luizote, Minas Gerais in Brazil; de Barros 2015). Nonetheless, we recommend further investigations covering more of the host spectrum and denser sampling for multiple hosts from different geographical regions to validate host association within the genus *Mastophorus*. The observed morphological and genetic variations distinguish isolates corresponding to different host usage that might justify separation into different species, if not genera. However, comparing species pairs, whether our results influence or motivate studies to advocate the change in taxonomy, goes beyond the scope of our work.

In addition to the morphological evidence, our study considers the information provided by the phylogeny with different marker genes, while the CO1 gene has been shown to be more informative because of the higher substitution rate (Blouin 2002) which results in a higher resolution of the CO1 tree. Comparing species pairs, Blouin reported genetic divergence of around 10% (range 6.9–13.0) for the CO1 gene (Blouin 2002). Thus, the *M. muris* specimens from *Mus* and *non-Mus* may be considered different species. In addition, ITS sequences distinguish between closely related parasitic nematode species and were consistent with both 18S and CO1 analyses; our *M. muris* isolates from house mice cluster in one clade in a sister group relation to the *Mastophorus* sequence from non-*Mus* hosts. Overall, our genetic analyses based on the three marker genes (18S, CO1 and ITS) support the subdivision of the genus *Mastophorus* in the two proposed variants with moderate to good support.

Considering the differentiation of members from the genera *Mastophorus* and *Protospirura*, our phylogenetic analyses suggest the separation as stated by previous morphological descriptions (Quentin et al. 1968; Quentin 1969). The phylogenetic tree based on ITS and CO1 supports the respective monophyly of the genus *Mastophorus*. Thus, based on the novel available genetic data, the relationship between the genera *Mastophorus* and *Protospirura* within the order Spirurida could be less controversial. The cosmopolitan *Protospirura muricola* is clearly separated from *Mastophorus* spp. based on the phylogeny provided here. The sequences from *P. muricola* built a separate group with CO1 sequence identities of 84.99–85.62% (472 bp) to *Mastophorus* from *Mus*. Misidentifications, erroneously assigned sequences like those from *P. numidica* sequences obtained from specimens collected from *Oxymycterus* in Brazil (de Barros 2015) assigned to the genus *Prostospirura* without describing details or illustrating their specimens, while belonging to the genus *Mastophorus* (Costa et al. 2018, 2022), are challenging to conclude and might promote confusion in the classification. While further studies should focus on data generation, validation (by associated taxonomic annotation) and phylogenetic analysis of reference sequences to clarify the confusion of *Mastophorus* with *Protospirura*, our work provides a deeper insight into the morphological and phylogenetic diversity of the genus *Mastophorus.*

### Supplementary Information

Below is the link to the electronic supplementary material.

Supplementary file1 (DOCX 2813 KB)

Supplementary file2 (DOCX 17 KB)

## Data Availability

Sequences obtained and used for the analysis are available at NCBI GenBank with the accession numbers: 18S [MN086286–MN086291], CO1 [MK867474–MK867480] and ITS [MK829001–MK829007]. Voucher specimens for *M. muris* from house mouse identified in this study were deposited in the Natural History Museum in Berlin, Germany in the department “Vermes” under specimens numbers E.7635–E.7639.
