# Supplementary material for: Dentition patterns and molecular diversity of Mastophorus muris (Gmelin, 1790) (Nematoda: Spiruroidea) support a host-associated subdivision
Source: Parasitol Res. 2024 Jun 10;123(6):237. doi: 10.1007/s00436-024-08259-1 (PMC11164724; doi:10.1007/s00436-024-08259-1)
Supplement: Supplementary file 1 — Supplementary file1 (DOCX 2813 KB) [file 436_2024_8259_MOESM1_ESM.docx]

Dentition patterns and molecular diversity of Mastophorus muris (Gmelin, 1790) (Nematoda: Spiruroidea) supports a host-associated subdivision.

Jost, Jenny; Hirzmann, Jörg; Ďureje, Ľudovít; Maaz, Denny; Martin, Peer; Stach, Thomas; Heitlinger, Emanuel and Jarquín-Díaz, Víctor Hugo

Corresponding author:

Víctor Hugo Jarquín-Díaz - vhjarquind@gmail.com / VictorHugo.JarquinDiaz@mdc-berlin.de

Max-Delbrück-Center for Molecular Medicine in the Helmholtz Association (MDC). Robert-Rössle-Str. 10, 13125 Berlin, Germany


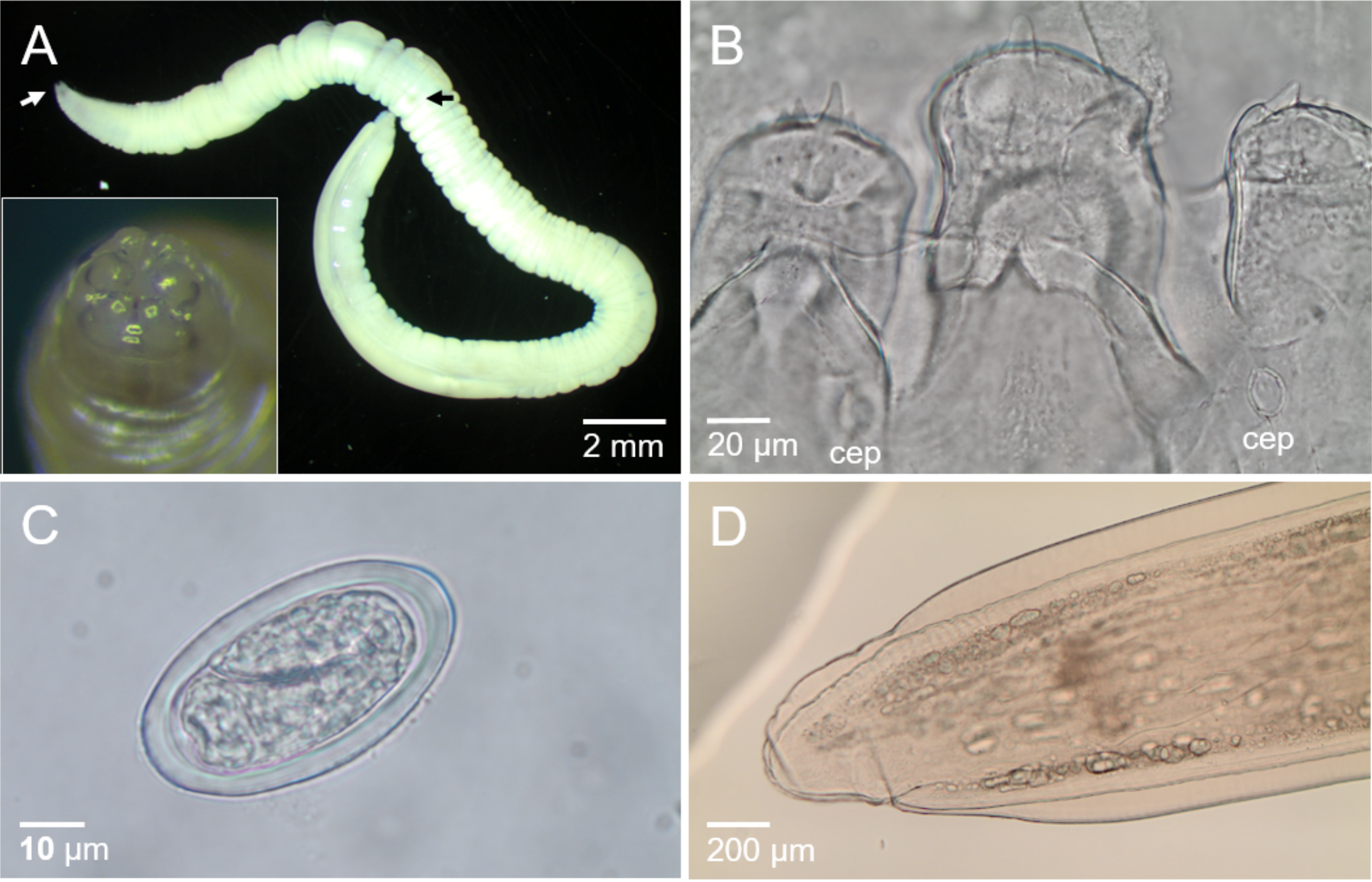
**Supplementary Figure S1:** Light micrographs of female *M. muris* specimen from *F. silvestris silvestris*. (A) Whole worm showing the vulva position (black arrow) in the first third of the body and the anterior end (white arrow) with prominent trilobed pseudolabia (detail in box). (B) Lobes of one pseudolabium with cephalic papillae (cep) and dentition on the inner membrane. (C) Thick-shelled egg with embryo obtained from female by pressure. (D) Tail.
