# Supplementary material for: Dentition patterns and molecular diversity of Mastophorus muris (Gmelin, 1790) (Nematoda: Spiruroidea) support a host-associated subdivision
Source: Parasitol Res. 2024 Jun 10;123(6):237. doi: 10.1007/s00436-024-08259-1 (PMC11164724; doi:10.1007/s00436-024-08259-1)
Supplement: Supplementary file 2 — Supplementary file2 (DOCX 17 KB) [file 436_2024_8259_MOESM2_ESM.docx]

**Supplementary Tables**

Jost, Jenny; Hirzmann, Jörg; Ďureje, Ľudovít; Maaz, Denny; Martin, Peer; Stach, Thomas; Heitlinger, Emanuel and Jarquín-Díaz, Víctor Hugo

Corresponding author:

Víctor Hugo Jarquín-Díaz - vhjarquind@gmail.com / VictorHugo.JarquinDiaz@mdc-berlin.de

Max-Delbrück-Center for Molecular Medicine in the Helmholtz Association (MDC). Robert-Rössle-Str. 10, 13125 Berlin, Germany

**Supplementary Table 1: Primer details used for PCR amplification of *M. muris* for different genetic regions.**

| **Primer name** | **Sequence (5'-3')** | **Gene region** | **Amplicon size [bp]** | **Reference** |
| --- | --- | --- | --- | --- |
| Nem_18S_F | CGCGAATRGCTCATTACAACAGC | 18S | ~815 | Floyd et al. (2005) |
| Nem_18S_R | GGGCGGTATCTGATCGCC |  |  |  |
| Mm527_F | CATTGGACGAGAACTGGGCTC | 18S | ~1.200 | Newly designed |
| Mm1514_R | TGGGACACCGTTGKTACATG |  |  |  |
| Nem_ITS+_NC5_F | GTAGGTGAACCTGCGGAAGGATCATT | ITS, 5.8S and ITS2 | ~1.100 | Gasser et al. (1995) |
| Nem_ITS+_NC2_R | TTAGTTTCTTTTCCTCCGCT |  |  |  |
| JB3 (NT-COI_239_F) | TTTTTTGGGCATCCTGAGGTTTAT | COI | ~359 | Bowles et al. (1993) |
| JB4.5 (NT-COI_240_R) | TAAAGAAAGAACATAATGAAAATG |  |  |  |
| NemCOIintF | TGATTGGTGGTTTTGGTAA | COI | ~650 | Casiraghi et al. (2001) |
| NemCOIintR | ATAAGTACGAGTATCAATATC |  |  |  |

**Supplementary Table 2: Composition of PCR reaction and cycler conditions for each primer pair.**

| **Primer name** | **Nem_18S** | | | **Mm527_1514** | | | **Nem_ITS+** | | | **NT_COI** | | | **NemCOIint** | | |
| --- | --- | --- | --- | --- | --- | --- | --- | --- | --- | --- | --- | --- | --- | --- | --- |
| Mastermix conditions [20µl] | Final concentration ; Volume [µl] | | | | | | | | | | | | | | |
| dH2O | - | 13.3 | | - | 12.9 | | - | 12.9 | | - | 11.7 | | - | 12.9 | |
| 10x DreamTaq Buffer | - | 2 | | - | 2 | | - | 2 | | - | 2 | | - | 2 | |
| dNTPS | 0.8 mM | 1.6 | | 0.5 mM | 1 | | 0.5 mM | 1 | | 0.5 mM | 1 | | 0.5 mM | 1 | |
| Forward primer | 0.5 µM | 1 | | 0.5 µM | 1 | | 0.5 µM | 1 | | 0.5 µM | 1 | | 0.5 µM | 1 | |
| Reverse Primer | 0.5 µM | 1 | | 0.5 µM | 1 | | 0.5 µM | 1 | | 0.5 µM | 1 | | 0.5 µM | 1 | |
| MgCl2 | - | 0 | | - | 0 | | - | 0 | | 1.5 mM | 1.2 | | - | 0 | |
| DreamTaq DNA Polymerase | 0.025 U/µl | 0.1 | | 0.025 U/µl | 0.1 | | 0.025 U/µl | 0.1 | | 0.025 U/µl | 0.1 | | 0.025 U/µl | 0.1 | |
| Template DNA | - | 1 | | - | 2 | | - | 2 | | - | 2 | | - | 2 | |
| Cycler conditions | Time [min]; Temperature [C°]; Cycles | | | | | | | | | | | | | | |
| Initial activation step | 05:00 | 94 |  | 05:00 | 95 |  | 01:00 | 94 |  | 10:00 | 95 |  | 03:00 | 95 |  |
| Denaturation step | 00:30 | 94 | 35 | 00:30 | 94 | 35 | 00:30 | 94 | 30 | 00:30 | 94 | 35 | 00:45 | 94 | 40 |
| Annealing | 00:30 | 54 |  | 00:30 | 51 |  | 00:30 | 55 |  | 00:30 | 50 |  | 00:45 | 52 |  |
| Extension | 01:00 | 72 |  | 01:00 | 72 |  | 00:30 | 72 |  | 00:30 | 72 |  | 01:30 | 72 |  |
| Final extension step | 10:00 | 72 |  | 10:00 | 72 |  | 07:00 | 72 |  | 10:00 | 72 |  | 10:00 | 72 |  |

**Supplementary Table 3: Nematode reference sequences from different gene regions available in NCBI used in phylogenetic analyses.**

| **Species** | **Host species** | **Location** | **Gene region** | **Lenght (bp)** | **GenBank accession no.** | **Reference** |
| --- | --- | --- | --- | --- | --- | --- |
| *Dirofilaria immitis* | *Culicidae sp.* | USA | 18S | 1329 | AF182647 | Watts et al. (1999) |
| *Dirofilaria repens dog* | *Canis familiaris* | India | 18S | 823 | MG780293 | Pradeep et al. (2018) |
| *Dirofilaria repens HS* | *Homo sapiens* | Japan | 18S | 1744 | AB973229 | Suzuki et al. (2015) |
| *Gongylonema neoplasticum* | *Rattus norvegicus* | Thailand | 18S | 1756 | LC331000 | Setsuda et al. (2018) |
| *Gongylonema pulchrum* | *Saimiri boliviensis* | Japan | 18S | 1756 | AB495401 | Halajian et al. (2010) |
| *Mastophorus muris rat* | *Rattus norvegicus* | South Africa | 18S | 1134 | MG356473 | Julius, et al. (2018) |
| *Mastophorus muris wildcat* | *Felis silvestris silvestris* | Germany | 18S | 1756 | MG818763 | Present study |
| *Mastophorus muris BER008* | *Apodemus flavicollis* | Germany | 18S | 779 | MN08629 | Present study |
| *Protospirura sp.* | *Mastomys coucha* | South Africa | 18S | 1677 | KY462830 | Julius, et al. (2018) |
| *Gongylonema sp. FE2012* | *Otus scops* | Spain | 18S | 699 | JX401925 | Esperon et al. (2013) |
|  |  |  |  |  |  |  |
| *Dirofilaria immitis* | *Canis familiaris* | Korea | COI | 441 | AF181893 | Lee et al. unpublished |
| *Dirofilaria immitis* | *Canis familiaris* | Australia | COI | 858 | AJ537512 | Hu et al. (2003) |
| *Dirofilaria repens HS* | *Homo sapiens* | Japan | COI | 715 | AB973225 | Suzuki et al. (2015) |
| *Gonyglynema neoplasticum* | *Rattus norvegicus* | Thailand | COI | 818 | LC331040 | Setsuda et al. (2018) |
| *Gongylonema pulchrum* | *Saimiri sciureus* | Japan | COI | 369 | AB513730 | Makouloutou et al. (2013) |
| *Mastophorus muris rat* | *Rattus norvegicus* | South Africa | COI | 398 | MG386206 | Julius, et al. (2018) |
| *Mastophorus muris BER008* | *Apodemus flavicollis* | Germany | COI | 804 | MK867480 | Present study |
| *Mastophorus muris wildcat* | *Felis silvestris silvestris* | Germany | COI | 858 | MG821081 | Present study |
| *Mastophorus muris Sigmodon* | *Sigmodon hispidus* | USA | COI | 710 / 710 / 655 | MH782850 / MH782851 /MH782852 | Thompson et al. (2019) |
| *Protospirura muricola* | *Gorilla sp.* | Central African Republic | COI | 591 | KP760207 | Lefoulon et al. (2015) |
| *Protospirura muricola* | *Gorilla gorilla* | Cameroon | COI | 474 | MN890122/ MN890123/ MN890124 | Gaillard et al., (2020) |
| *Protospirura sp.* | *Cercopithecus nictitans* | Cameroon | COI | 474 | MN890114/ MN890093/ MN890094 | Gaillard et al., (2020) |
|  |  |  |  |  |  |  |
| *Dirofilaria immitis* | *Canis familiaris* | Taiwan | ITS | 1132 | AF217800 | Mar et al. (2002) |
| *Dirofilaria immitis solgi* | *Canis familiaris* | Iran | ITS | 1132 | KY863453 | Solgi et al. (2018) |
| *Dirofilaria repens HS* | *Homo sapiens* | Japan | ITS | 1351 | AB973229 | Suzuki et al. (2015) |
| *Dirofilaria repens DR2* | NA | Thailand | ITS | 560 | AY621480 | Nuchprayoon et al. unpublished |
| *Gonyglynema neoplasticum* | *Rattus norvegicus* | Thailand | ITS | 1351 | LC331000 | Setsuda et al. (2018) |
| *Gongylonema pulchrum* | *Saimiri boliviensis* | Japan | ITS | 1351 | AB495401 | Halajian et al. (2010) |
| *Mastophorus muris wildcat* | *Felis silvestris silvestris* | Germany | ITS | 1351 | MG818763 | Present study |
| *Protospirura sp. S19* | *Rattus rattus* | Spain | ITS | 892 | JF514771 | Feliu, C. unpublished |
| *Protospirura sp. S17* | *Rattus rattus* | Spain | ITS | 888 | JF514769 | Feliu, C. unpublished |
| *Protospirura sp. S45* | *Rattus rattus* | Spain | ITS | 892 | JF514770 | Feliu, C. unpublished |
| **References (In order of appearance)** |  |  |  |  |  |  |
| Watts, K. J., Courtney, C. H., & Reddy, G. R. (1999). Development of a PCR-and probe-based test for the sensitive and specific detection of the dog heartworm, Dirofilaria immitis, in its mosquito intermediate host. Molecular and cellular probes, 13(6), 425-430. | | | | | | |
| Pradeep, R. K., Nimisha, M., Pakideery, V., Johns, J., Chandy, G., Nair, S., ... & Ravindran, R. (2019). Whether Dirofilaria repens parasites from South India belong to zoonotic Candidatus Dirofilaria hongkongensis (Dirofilaria sp. hongkongensis)?. Infection, Genetics and Evolution, 67, 121-125. | | | | | | |
| Suzuki, J., Kobayashi, S., Okata, U., Matsuzaki, H., Mori, M., Chen, K. R., & Iwata, S. (2015). Molecular analysis of Dirofilaria repens removed from a subcutaneous nodule in a Japanese woman after a tour to Europe. Parasite, 22. | | | | | | |
| Setsuda, A., Ribas, A., Chaisiri, K., Morand, S., Chou, M., Malbas, F., ... & Sato, H. (2018). Molecular genetic diversity of Gongylonema neoplasticum (Fibiger & Ditlevsen, 1914)(Spirurida: Gongylonematidae) from rodents in Southeast Asia. Systematic parasitology, 95(2), 235-247. | | | | | | |
| Halajian, A., Eslami, A., Salehi, N., Ashrafi-Helan, J., & Sato, H. (2010). Incidence and genetic characterization of Gongylonema pulchrum in cattle slaughtered in Mazandaran Province, northern Iran. Iranian journal of parasitology, 5(2), 10. | | | | | | |
| Julius, R. S., Schwan, E. V., & Chimimba, C. T. (2018). Molecular characterization of cosmopolitan and potentially co-invasive helminths of commensal, murid rodents in Gauteng Province, South Africa. Parasitology research, 117(6), 1729-1736. | | | | | | |
| Lefoulon, E., Bain, O., Bourret, J., Junker, K., Guerrero, R., Cañizales, I., ... & Martin, C. (2015). Shaking the tree: multi-locus sequence typing usurps current onchocercid (filarial nematode) phylogeny. PLoS neglected tropical diseases, 9(11), e0004233. | | | | | | |
| Perera, A., Maia, J. P. M. C., Jorge, F., & Harris, D. J. (2013). Molecular screening of nematodes in lacertid lizards from the Iberian Peninsula and Balearic Islands using 18S rRNA sequences. Journal of Helminthology, 87(2), 189. | | | | | | |
| Esperón, F., Martín, M. P., Lopes, F., Orejas, P., Carrero, L., Muñoz, M. J., & Alonso, R. (2013). Gongylonema sp. infection in the scops owl (Otus scops). Parasitology international, 62(6), 502-504. | | | | | | |
| Hu, M., Gasser, R. B., El-Osta, Y. A., & Chilton, N. B. (2003). Structure and organization of the mitochondrial genome of the canine heartworm, Dirofilaria immitis. Parasitology, 127(1), 37. | | | | | | |
| Makouloutou, P., Setsuda, A., Yokoyama, M., Tsuji, T., Saita, E., Torii, H., ... & Sato, H. (2013). Genetic variation of Gongylonema pulchrum from wild animals and cattle in Japan based on ribosomal RNA and mitochondrial cytochrome c oxidase subunit I genes. Journal of helminthology, 87(3), 326. | | | | | | |
| Thompson, A. T., Cleveland, C. A., Koser, T. M., Wyckoff, S. T., & Yabsley, M. J. (2019). The Occurrence of Physaloptera Hispida and a Mastophorus Sp. in Pulmonary Vessels of Hispid Cotton Rats (Sigmodon hispidus) from Georgia, USA. The Journal of parasitology, 105(5), 718-723. | | | | | | |
| Gaillard, C. M., Pion, S. D., Hamou, H., Sirima, C., Bizet, C., Lemarcis, T., ... & Locatelli, S. (2020). Detection of DNA of filariae closely related to Mansonella perstans in faecal samples from wild non-human primates from Cameroon and Gabon. Parasites & vectors, 13(1), 1-13. | | | | | | |
| Mar, P. H., Yang, I. C., Chang, G. N., & Fei, A. C. Y. (2002). Specific polymerase chain reaction for differential diagnosis of Dirofilaria immitis and Dipetalonema reconditum using primers derived from internal transcribed spacer region 2 (ITS2). Veterinary parasitology, 106(3), 243-252. | | | | | | |
| Solgi, R., Sadjjadi, S. M., Mohebali, M., Zarei, Z., Golkar, M., & Raz, A. (2018). Development of new recombinant DgK antigen for diagnosis of Dirofilaria immitis infections in dogs using ELISA technique and its comparison to molecular methods. Iranian biomedical journal, 22(4), 283. | | | | | | |

**Supplementary Table 4: Dentition pattern per sample observed by SEM.**

| **Sample ID** | **Sex** | **Trilobed pseudolabia** | | | | | | | | | | | | | | |
| --- | --- | --- | --- | --- | --- | --- | --- | --- | --- | --- | --- | --- | --- | --- | --- | --- |
|  |  | **left** | | | | | **middle** | | | | | **right** | | | | |
| AA_0256f_7 | F | nv | nv | nv | nv | nv | nv | nv | nv | nv | nv | nv | nv | nv | nv | nv |
|  |  | nv | nv | nv | nv | nv | nv | nv | nv | nv | nv | nv | nv | nv | nv | nv |
| AA_0256m_8 | M | nv | 2 | 1 | nv | nv | 1 | 5 | 1 | 4 | 1 | nv | 2 | 1 | 3 | 1 |
|  |  | nv | 2 | 1 | nv | nv | 1 | 4 | 1 | 3 | 1 | nv | nv | 1 | 2 | 1 |
| AA_0348f_9 | F | nv | 3 | nv | nv | nv | 1 | 3 | 1 | 4 | 1 | nv | nv | nv | nv | nv |
|  |  | nv | nv | 1 | nv | nv | 1 | 2 | 1 | 3 | 1 | nv | nv | 1 | 3 | nv |
| AA_0348m_10 | M | nv | 2 | 1 | 2 | 1 | nv | 2(+) | 1 | 5 | 1 | nv | nv | 1 | nv | nv |
|  |  | nv | nv | nv | nv | nv | 1 | 4 | 1 | 2(+) | 1 | nv | nv | 1 | nv | nv |
| AA_0351f_11 | F | nv | 2 | 1 | nv | nv | 1 | 3 | 1 | 3 | 1 | 1 | nv | 1 | 2 | 1 |
|  |  | nv | 2 | 1 | 3 | nv | 1 | 4 | 1 | 4 | 1 | 1 | 3 | 1 | 2 | 1 |
| AA_0351m_12 | M | 1 | 2 | 1 | nv | nv | 1 | nv | 1 | nv | 1 | nv | nv | 1 | nv | nv |
|  |  | nv | nv | 1 | 3 | nv | 1 | nv | 1 | nv | 1 | nv | nv | nv | 2 | nv |
| AA_0349f_6 | F | nv | nv | nv | nv | nv | 1 | 5 | 1 | nv | | | | 1 | 2(+) | nv |
|  |  | nv | nv | 1 | nv | nv | 1 | 3 | 1 | 2 | 1 | 1 | nv | 1 | 3 | nv |
| AA_0349m_7 | M | nv | nv | 1 | 1(+) | nv | 1 | 3 | 1 | 2 | 1 | nv | nv | 1 | 2 | 1 |
|  |  | nv | nv | 1 | nv | 1 | 1 | 4 | 1 | 3 | 1 | nv | 3 | 1 | 2 | nv |
| AA_0506f_8 | F | nv | 1(+) | nv | 2(+) | 1 | 1 | 5 | 1 | 4 | 1 | nv | nv | 1 | 2 | nv |
|  |  | 1 | 2 | 1 | nv | nv | nv | nv | 1 | 4 | 1 | 1 | 2 | 1 | 2 | 1 |
| AA_0348A | F | 1 | 3 | 1 | 3 | nv | 1 | 3 | 1 | 5 | 1 | nv | nv | 1 | 1(+) | nv |
|  |  | nv | 2(+) | 1 | nv | nv | nv | 2(+) | 1 | 2(+) | nv | nv | nv | nv | 2 | 1 |
| AA_0348C | F | 1 | nv | 1 | nv | nv | nv | nv | nv | nv | 1 | nv | nv | 1 | nv | nv |
|  |  | nv | nv | 1 | nv | nv | 1 | 4 | 1 | 2(+) | nv | nv | nv | nv | nv | nv |
| AA_0256D | F | 1 | 2 | 1 | nv | nv | nv | 4 | 1 | 2(+) | nv | nv | nv | 1 | 2 | 1 |
|  |  | nv | 2 | 1 | 2 | nv | 1 | 5 | 1 | 3 | nv | 1 | 2 | 1 | 3 | nv |
|  |  | 1 | (2-3) | 1 | (2-3) | 1 | 1 | (2-5) | 1 | (2-5) | 1 | 1 | (2-3) | 1 | (2-3) | 1 |
|  |  |  |  |  |  |  |  |  |  |  |  |  |  |  |  |  |
| Mm_005_MG | F | nv | 1(+) | 1 | nv | nv | nv | 7 (3-1-3) | | | nv | nv | 1(+) | 1 | 2 | nv |
|  |  | nv | 2 | 1 | nv | nv | nv | 7(3-1-3) | | | nv | nv | nv | 1 | nv | nv |
| Mm_005_MG | M | nv | 2 | 1 | 3 | nv | nv | 9 (4-1-4) | | | nv | nv | 3 | 1 | 2 | nv |
|  |  | nv | 2(+) | 1 | 3 | nv | nv | 7 (2-1-4) | | | nv | nv | nv | 1 | 2 | nv |
|  |  |  | (2-3) | 1 | (2-3) |  |  | (2-4) | 1 | (2-4) |  |  | (2-3) | 1 | (2-3) |  |
|  |  |  |  |  |  |  |  |  |  |  |  |  |  |  |  |  |
| Mm_008_AF | F | nv | nv | nv | nv | nv | nv | nv | nv | nv | nv | nv | nv | nv | nv | nv |
|  |  | nv | nv | nv | nv | nv | nv | nv | nv | nv | nv | nv | nv | nv | nv | nv |
| Mm_008_AF | M | NA | NA | NA | NA | NA | NA | NA | NA | NA | NA | NA | NA | NA | NA | NA |
|  |  | NA | NA | NA | NA | NA | NA | NA | NA | NA | NA | NA | NA | NA | NA | NA |
|  |  | NA | | | | | | | | | | | | | | |

Each row represents results for one specimen with two trilobed pseudolabia. Host origin of specimens is specified as follows: AA – *Mus* , AF – *Apodemus* , MG- *Myodes.* F- Female, M- Male, nv - not visible, NA- not available.
